# Evaluating carbon stocks in soils of fragmented Brazilian Atlantic Forests (BAF) based on soil features and different methodologies

**DOI:** 10.1038/s41598-024-60629-y

**Published:** 2024-05-01

**Authors:** Iraê Amaral Guerrini, Jaqueline Pinheiro da Silva, Deicy Carolina Lozano Sivisaca, Felipe Góes de Moraes, Celso Anibal Yaguana Puglla, Carlos de Melo Silva Neto, Rafael Barroca Silva, Sérvio Túlio Pereira Justino, Ludmila Ribeiro Roder, Jason Nathaniel James, Gian Franco Capra, Antonio Ganga

**Affiliations:** 1 Department of Forest, Soil and Environmental Sciences, College of Agricultural Sciences, São Paulo State University-UNESP, Botucatu, SP 18610-034 Brazil; 2 Federal Institute of Education, Science and Technology of Goiás, Center of Reference in Research and Innovation – CITELAB IFG, Goiânia, GO 74594111 Brazil; 3 Dipartimento di Architettura, Design e Urbanistica, Università degli Studi di Sassari, Viale Piandanna No 4, 07100 Sassari, Italy; 4 Exponent, Inc., 15375 SE 30th Place, Bellevue, WA 98007 USA

**Keywords:** Forest ecology, Environmental impact

## Abstract

Brazil’s Atlantic Forest (BAF) is a highly fragmented, strategic environmental and socio-economic region that represents the fourth biodiversity hotspot while also producing many commodities that are exported globally. Human disturbance plays a pivotal role as a driver of BAF’s soil dynamics and behaviors. The soils under Late Primary and Secondary Semideciduous Seasonal Forests (LPSF and LSSF) were characterized by high to moderate resilience, with improved chemical properties as human disturbance decreased. The Transitional Forest to *Cerrado* (TFC) had the worst soil conditions. Disturbed Primary and Secondary Semideciduous Seasonal Forests (DPSF and DSSF) represent a transitional stage between LPSF/LSSF and TFC. Accordingly, SOCs stocks increased from TFC << DPSF, DSSF < LPSF, LSSF. In BAF soils, to avoid unreliable data, SOCs measurements should be (i) conducted to at least 1 m soil depth and (ii) quantified with a CHN analyzer. Human disturbance strongly affected the positive feedback between vegetation succession, SOCs, and soil nutrition. Soil development decreased as human disturbance increased, thus negatively affecting SOCs. Soils in the BAF require a long time to recover after the end of human disturbance, thus suggesting that preservation strategies should be prioritized in remnant BAF fragments.

## Introduction

After the oceans, the soil is the main carbon sink on our planet, storing about 2500 Pg C in the first 100 cm depth^1^, a value corresponding to three times the carbon stock in the atmosphere and about four and a half times the stock in the biosphere^2^.

As first reviewed by Harrison et al.^3^, most studies assess soil organic carbon stocks (SOCs) in the first 0–20/30 cm. The Intergovernmental Panel on Climate Change^4–6^ (IPCC) suggests sampling the first 0–30 cm to assess SOCs. Even if the IPCC encourages practitioners to take samples down to 1 m, the minimum required depth of 30 cm is the depth most frequently investigated worldwide. Indeed, such a depth is usually preferred for inventory and practical purposes, since it is often difficult, expensive, and time-consuming to collect soil samples in deeper soil horizons^3,7^. However, such a “minimum depth” represents only part of the overall carbon stock present in the soil^7^. Even if not restricted to tropical environments^8^, soils can reach impressive depth due to a combination of age, high precipitation, and/or repeated or thick deposition of unconsolidated parent materials, all of which can result in substantial underestimation of SOC when sampling is limited to the first 30 cm^9^. Previous investigations^10,11^ already demonstrated that a significant accumulation of SOC can occur in deeper soil horizons. For instance, Canary et al.^12^ and Liebig et al.^13^ compared the C stocks in the first 20/30 cm of mineral soil surface horizons (usually A or Ap) with the 20/30–100 cm soil depth; they outlined that the 0–20/30 cm soil contained an average of 44% of SOC *vs* 56% in deeper horizons. Such results show the inaccuracy of only sampling the first 20/30 cm soil surface if we want to assess the total SOC pool^14^. Another clear example of the bias introduced by only sampling surface soil came from an analysis of 2700 soil profiles in three global databases investigating (i) shrubland, (ii) grassland, and (iii) forest ecosystems. In this case, when the 0–20/30 cm were compared to 20/30–100 soil depth, authors observed 42% *vs* 58%, 33% *vs* 77%, and 50% *vs* 50% of the total SOC, respectively^15,16^.

The most commonly used technique for laboratory SOC measurement^17–19^ is the wet oxidation method by Walkley–Black^20^ (WBm, hereafter). This method determines SOC content by oxidizing SOC with a potassium dichromate (K_2_Cr_2_O_7_)-sulfuric acid (H_2_SO_4_) mixture followed by back titration of excess dichromate. However, there are several issues with the WBm method. First, the method does not result in complete soil organic matter (SOM) oxidation, causing an underestimation of total SOC content^21^. To avoid such a problem, a “correction factor”, which ranges greatly according to soil features, is applied according to investigated pedo-environmental conditions^19^. Second, dichromate is a carcinogenic compound and thus laboratory use generates toxic wastes that are hazardous to human health and the environment and require complex and expensive management and disposal^19^.

More accurate measurements can be done using an elemental analyzer, such as a CHN analyzer (CHNa, hereafter), which can detect all SOC forms in the analyzed soil samples. Additionally, the method is safe, repeatable, and can be automated while avoiding hazardous reagents. However, widespread use of CHNa in soil laboratories worldwide is limited by the high up-front costs of the machine as well as maintenance expenses^22^. This is a dramatic situation since reliable soil organic carbon measurement is pivotal in studies of greenhouse gas (GHG) emissions caused by human activities and responsible for global warming and climate change processes^23^. Thus, solutions to encourage and promote its worldwide adoption should be proposed.

Most SOC measurements in the literature come from a few countries, which over-represents those regions in our scientific understanding of human disturbance effects on soils and ecosystems^24^. At the continental scale, the imbalance in study density is stark^25,26^: (i) studies conducted in Asia, Europe, and North America represent 90% of global measurements; (ii) in Africa and South America, reliable data is dramatically lacking. Nonetheless, soils and ecosystems in Africa and South America have great significance to worldwide GHG budgets^19^.

Another pivotal question is related to the fact that soil physical and chemical properties strongly influence SOCs^27^. Thus, an in-depth soil analysis can avoid bias in SOCs assessment^21^. As argued by several scholars^28–31^, soil physical–chemical properties, such as soil depth and bulk density greatly influence SOCs. Other studies focused their attention on other soil properties such as macro- and micronutrient content and behavior ^22–34^. However, most of these studies were conducted on (i) soil surface horizons only and/or by (ii) taking into account only a few soil parameters, with no information on the whole complex relationships between multiple soil physical–chemical properties and SOCs in deep Tropical soils.

Brazil is one of the largest emerging economies. It is the tenth-largest world economy and the biggest within Latin American countries, reaching a gross domestic product (GDP) of nearly 8% immediately before the pandemic period (+ 1.9% in 2023^35^). The agribusiness and forestry sectors played a pivotal role in the historical expansion of the Brazilian economy, and output from these sectors continues to grow at substantial rates^36^. However, the same sector is responsible for important and large-scale land-use change, which affects soil C stocks and, consequently, GHG emissions^37^.

This strong relationship between the Brazilian economy and the agroforestry sector brought an extraordinary increase in agreements and collaborations among private companies and research centers (Universities, Governmental Bodies, etc.), which promoted development in soil laboratory technologies. Subsequently, tools such as elemental analyzers (e.g., CHNa) are now commonly used and widespread in the country. This represents an important opportunity to fill the knowledge gap still affecting the literature on C soil content in Brazilian soils, a unique pedoecosystem worldwide. This can also allow comparisons between different SOC stock assessment methods.

Brazil’s Atlantic Forest (BAF, hereafter) is a strategic biodiversity hotspot^36^ and is one of the most species-rich ecosystems on Earth^37^. Despite being considered under threat due to human-related degradation, it still represents the fourth most biodiverse hotspot in the world and is Brazil’s second-most diverse ecosystem (after the Amazon Forest)^38^. After European colonization (1500 AD), the original 130 million hectares of BAF^39^ were drastically destroyed and reduced, now hosting around 70% of Brazil’s population^36^. Only 26–28% of BAF continuous cover remains, most of which is in different stages of regeneration^27^.

The BAF region is also pivotal in the Brazilian and worldwide agroeconomy^35^. The agribusiness sector based in the BAF region is the global leader for the production of many commodities (such as coffee, corn, ethanol, meat, soybean, sugarcane, etc.), which are exported around the world and which make Brazil’s agroindustry the “breadbasket of the world”^40^. About 80% of the Brazilian Gross Domestic Product (GDP) is based on BAF region activities^35^. Yet, studies on the remaining native BAF can bring new knowledge and opportunities to reconcile conservation and ecosystem services purposes *vs* socio-economic activities. This represents an enormous challenge that science must face to safeguard this important, still understudied ecosystem^27^, trying, when possible, to allow the economic development of an area that produces essential goods. Among these challenges, estimating C stocks stored in these unique and fragile pedosystems represents a starting point of knowledge on which to make future choices of how and in which way to reconcile ecosystem protection and socio-economic development. Indeed, the soil degradation historically affecting BAF causes drastic changes in their bio-physical–chemical properties, negatively affecting fertility, nutrient/water storage availability, and globally, being responsible for GHG emissions^41^. On a global scale, deforestation is estimated to contribute 8–15% of annual GHG emissions^42^. In Brazil, which is the world’s fifth-largest GHG emitter, the leading causes of CO_2_ emissions are changes in land use, representing 75% of the total emissions *vs* the 20–25% coming from burning fossil fuels^43^. Therefore, there is a need to better understand, by using and comparing advanced *vs* commonly used tools of measurements, the SOC sequestration in tropical pedosystems, aiming to outline conservation and management policies for reducing CO_2_ emissions in a critical ecosystem.

Overall, taking into account all the previous considerations, this research aimed to: (i) quantify C stocks in BAF soils by (ii) comparing WBm vs CHNa methods, along (iii) different soil pedosystems (investigated to 1 m soil depth) featuring (iv) five different BAF fragments (in terms of human-disturbance magnitude) belonging to one of the most important, human-affected, and fragile ecosystem worldwide. A full suite of soil physical and chemical analyses was conducted in addition to SOC quantification to understand (vi) how soil features and different methodologies influence SOCs assessment. The present paper first combined and focused on all these related and pivotal aspects in one single research.

## Results

### Soil physical–chemical features

Table 1 shows selected soil physical–chemical features of all five investigated BAF fragments—from least to most disturbed, Late Primary and Secondary Semideciduous Seasonal Forests (LPSF and LSSF), Disturbed Primary and Secondary Semideciduous Seasonal Forests (DPSF and DSSF), and Transitional Forest to *Cerrado* (TFC)—along five different depths (0–20 to 80–100 cm). The mean pH-CaCl_2_ value was 4.9 ± 0.1, ranging from 3.4 (very acidic) to 6.9 (neutral). Inside each investigated area, pH usually significantly decreased (p < 0.05) with depth, except for TFC, where it maintained similar low (acidic) values. Comparing different fragments, pH decreased from LPSF, LSSF > DPSF, DSSF > TFC. The BD ranged from very low (0.6) up to extremely high (1.9 g cm^−3^) with a mean of 1.3 ± 0.1 g cm^−3^. As expected, BD increased with soil depth in all investigated BAF fragments. In this case, a decreasing trend from TFC > DPSF, DSSF > LPSF, LSSF was observed among environments. Soil organic matter (SOM) and macronutrients N and P concentrations had variable contents too, with usually low mean values of 19.0 ± 0.9 g kg^−1^ (min: 0.1; max: 86.3 g kg^−1^), 0.3 ± 0.1% (0.1–3.2%), and 30.1 ± 2.2 mg kg^−1^ (min: 1.6; max: 546.2 mg kg^−1^), respectively. Their values generally decreased along the soil depth, showing the same pattern when compared to BAF fragments (LPSF, LSSF > DPSF, DSSF > TFC). Cation-exchange capacity (CEC: mean 109.1 ± 3.4 mmol_c_ dm^−3^, range 24.0–381.6 mmol_c_ dm^−3^), exchangeable basic cations (K^+^: 1.8 ± 0.2 mmol_c_ dm^−3^, 0.2–7.8 mmol_c_ dm^−3^; Ca^2+^: 57.2 ± 2.8 mmol_c_ dm^−3^, 2.4–297.0 mmol_c_ dm^−3^; Mg^2+^: 15.4 ± 0.6 mmol_c_ dm^−3^, 0.6.0–43.2 mmol_c_ dm^−3^), and, consequently, base saturation (BS: 74 ± 3%, 3–343%), showed a strict positive relationship with SOM (vide infra) and all exchangeable basic cations, while acidic cations (Al^3+^: 3.4 ± 0.3 mmol_c_ dm^−3^, 0.0–57.0 mmol_c_ dm^−3^) and total acidity (H + Al: 34 ± 1.3 mmol_c_ dm^−3^, 5.9–296.7 mmol_c_ dm^−3^) had a negative relationship with SOM. Thus, CEC and all basic cations usually decreased along soil depth, while the opposite was usually observed for Al^3+^ and H + Al. Comparing the five BAF fragments, the trend DPSF, DSSF > LPSF, LSSF >> TFC was observed for CEC and all basic cations, while TFC > DPSF, DSSF ≃ LPSF, LSSF was observed for Al^3+^ and H + Al.

**Table 1:** Soil selected physical–chemical properties in the investigated BAF's fragments (mean ± *SE*).

Depth	pH	BD	SOM	N CHN	P	Al^3+^	H + Al	K^+^	Ca^2+^	Mg^2+^	CEC	BS
cm		g cm^−3^	g kg^−1^	%	mg kg^−1^	mmol_c_ dm^−3^						%
Late Primary Semideciduous Seasonal Forest (LPSF)												
0–20	5.8aA 0.2	0.9aA 0.0	69.5aA 4.4	0.53aA 0.0	64.2bA 8.1	0.17bA 0.1	33.9aA 5.9	2.4aA 0.4	116.3aA 0.5	28.5aA 0.3	181.2aA 5.7	147aA 1
20–40	5.8aA 0.1	1.0aA 0.0	56.7aA 5.4	0.45aA 0.1	69.2aA 11.4	0.16bA 0.05	31.6aA 4.2	2.3bA 0.5	113.9aA 2.3	28.9aA 0.2	176.6aA 4.8	145aA 3
40–60	5.6bA 0.2	1.1bA 0.0	40.9bA 6.2	0.35bA 0.07	64.9bAB 14.6	0.22cA 0.05	36.0aA 4.4	2.2cA 0.7	111.3bA 5.3	27.9bA 0.6	177.5aA 7.5	141aA 6
60–80	5.6bA 0.3	1.2b 0.0	33.5cA 6.4	0.43cA 0.04	43.0cB 8.8	0.63aA 0.46	32.5aA 7.0	1.9dA 0.7	89.8cA 11.9	29.0bA 0.6	153.3bA 6.1	120bA 12
Late Secondary Semideciduous Seasonal Forest (LSSF)												
0–20	6.2aA 0.3	0.9aA 0.0	63.8aA 6.3	0.52aA 0.02	72.1aA 8.3	0.00cB 0.00	25.6aA 6.5	5.9bB 0.7	104.5aA 7.2	26.3aA 1.9	162.3aA 2.3	137aA 7
20–40	6.2aA 0.3	0.9aA 0.1	49.7bA 3.7	0.41bA 0.06	62.0bA 11.2	0.16bA 0.09	26.8aA 8.3	6.4aB 0.5	100.3aA 10.5	24.8aA 2.7	158.2aB 5.6	131aB 10
40–60	6.0bA 0.5	1.1bA 0.0	42.4bA 8.2	0.46aA 0.06	55.5bAB 10.2	0.11bB 0.01	32.6bA 10.8	6.4aB 0.6	94.1bA 10.6	22.7bA 2.7	155.8aB 5.2	123aB 10
60–80	5.7cA 0.4	1.2cA 0.0	30.7cA 4.4	0.44aA 0.05	45.4cB 13.0	0.37aB 0.24	31.6bA 7.8	6.3aB 1.1	78.2cA 13.4	19.7cB 2.9	135.8bB 14.2	104bB 14
Disturbed Primary Semideciduous Seasonal Forest (DPSF)												
0–20	5.4aB 0.1	1.1aB 0.0	28.2aB 2.6	0.40aB 0.03	30.2aB 4.3	0.53dC 0.09	22.8aA 1.1	3.0aAB 0.2	74.4aB 7.7	17.8aB 1.4	118.0aB 9.0	95aB 9
20–40	5.0bB 0.1	1.3bB 0.0	17.7bB 2.0	0.31bB 0.02	22.7bB 3.6	1.40cC 0.30	29.0bA 2.2	1.7bAB 0.2	61.4aB 8.5	15.3bB 1.6	107.2bC 10.4	78bC 10
40–60	5.0bB 0.1	1.3bB 0.0	12.7cB 1.8	0.27bB 0.02	23.2bC 4.2	2.18bC 0.66	31.9bA 4.1	1.4bAB 0.1	55.3aB 8.7	15.0cB 1.7	103.5bC 11.0	72bC 10
60–80	4.9bB 0.1	1.4cB 0.0	9.0dB 1.2	0.31bB 0.06	21.1bC 4.2	3.35aC 1.14	35.8cA 5.6	1.3bAB 0.1	54.7aB 9.6	15.6bB 1.8	107.4bC 12.0	72bC 11
80–100	4.9bA 0.1	1.5dA 0.0	8.6dA 1.3	0.26bA 0.02	31.1aA 11.2	3.79aA 1.19	36.7cA 5.4	1.4cA 0.2	53.1aA 9.4	16.3aA 1.8	107.5bA 11.7	71bA 11
Disturbed Secondary Semideciduous Seasonal Forest (DSSF)												
0–20	4.9aB 0.2	1.2aB 0.0	27.1aB 4.9	0.38aB 0.03	60.2aAB 23.2	1.87dD 0.65	38.8aB 4.5	2.0aA 0.2	65.3aB 11.1	18.5aB 2.7	124.5aB 13.2	86aB 14
20–40	4.6bB 0.2	1.3aB 0.0	16.8bB 3.7	0.29aB 0.02	42.4bAB 11.8	3.38aD 1.21	48.44bB 5.97	1.2bA 0.1	55.6aB 11.1	16.4aB 2.9	121.6aC 14.3	73aC 14
40–60	4.5bB 0.2	1.4abB 0.0	11.5cB 2.8	0.27bB 0.02	45.0bAC 16.0	4.39aD 1.40	44.0abB 5.9	0.9cA 0.1	50.1aB 10.7	15.1bB 3.2	110.0abC 15.0	66abC 14
60–80	4.4bB 0.3	1.5bcB 0.0	4.2dC 1.3	0.25cB 0.03	21.7cAC 9.4	7.13cD 2.54	41.7abB 9.6	0.7cA 0.1	29.4bC 10.1	8.4cC 3.3	80.1bD 13.4	38bD 13
80–100	4.4bA 0.3	1.5bcA 0.0	3.3eB 0.9	0.22dA 0.02	23.7cB 9.0	8.60bB 3.5	55.6cB 17.4	0.7bcB 0.2	32.2bB 10.0	8.9cB 3.4	97.5bB 17.0	42bB 13
Transitional Forest to *Cerrado* (TFC)												
0–20	3.7aC 0.1	1.3aC 0.0	17.5aC 1.1	0.19aC 0.03	6.2aC 0.5	9.79bE 1.20	56.7aC 5.9	1.0aC 0.1	5.4aC 1.0	3.3aC 0.5	66.5aC 5.3	10aC 2
20–40	3.7aC 0.1	1.4abC 0.0	10.8bC 0.8	0.17aC 0.02	3.3bC 0.3	11.44aE 0.89	47.6abB 3.6	0.6aC 0.1	3.6bC 0.6	1.3bC 0.2	53.2abC 3.3	6abD 1
40–60	3.8abC 0.0	1.5bC 0.0	7.4cC 0.4	0.16aC 0.02	2.4bC 0.12	12.06aE 0.58	35.5bA 1.1	0.3bC 0.0	2.4cC 0.0	1.0cC 0.1	39.1bC 1.1	4bD 0
60–80	3.9bC 0.0	1.5bB 0.0	6.0cBC 0.4	0.17aC 0.02	2.3bC 0.05	11.30aE 0.61	32.07bA 1.11	0.2bC 0.0	2.4cC 0.0	0.9cC 0.1	35.5bC 1.1	3bE 0
80–100	3.8abB 0.0	1.5bA 0.0	5.7cC 0.6	0.15aB 0.02	2.3bC 0.1	12.38aC 0.60	37.5bA 1.7	0.2bC 0.0	2.4cC 0.0	0.9cC 0.1	41.0bC 1.7	4bC 0

Different lowercase letters along columns (inside the same BAF fragments) showed significant statistical differences for different soil depths at p < 0.05. Different capital letters along columns (among the five different BAF fragments) showed significant statistical differences for the same soil horizons at p < 0.05).

Soil S and micronutrients (Table 2) broadly varied (S: 9.79 ± 0.29 mg kg^−1^, 1.57–51.68 mg kg^−1^; B: 0.32 ± 0.00 mg kg^−1^, 0.05–1.39 mg kg^−1^; Cu: 3.11 ± 0.15 mg kg^−1^, 0.11–13.68 mg kg^−1^; Fe: 56.26 ± 1.94 mg kg^−1^, 1.78–170.69 mg kg^−1^; Mn: 28.22 ± 1.30 mg kg^−1^, 0.64–104.0 mg kg^−1^; Zn: 8.76 ± 0.48 mg kg^−1^, 0.16–57.72 mg kg^−1^) usually showing a decreasing trend with soil depth (except Cu and Zn) and decreased across environments from LPSF, LSSF > DPSF, DSSF > TFC.

**Table 2:** Soil S and micronutrients content in the investigated BAF’s fragments (mean ± *SE*).

Depth	S	B	Cu	Fe	Mn	Zn
cm	mg kg^−1^					
Late Primary Semideciduous Seasonal Forest (LPSF)						
0–20	17.50aA 1.19	0.55aA 0.06	4.77cA 0.43	78.71aA 18.45	86.45aA 4.70	10.21aA 1.26
20–40	15.35aA 1.57	0.37bA 0.04	5.69bA 0.44	76.68aA 16.71	85.64aA 11.55	14.50bA 1.86
40–60	13.87bA 0.69	0.30bA 0.04	5.96aA 0.56	67.85bA 12.74	75.45cA 6.94	17.43cA 3.04
60–80	13.14bA 2.2	0.26bA 0.0	5.31bA 0.95	33.20cA 3.93	81.64bA 6.97	12.19aA 2.46
Late Secondary Semideciduous Seasonal Forest (LSSF)						
0–20	12.29aB 1.62	0.76aB 0.07	10.01aB 0.85	37.36aB 10.20	74.31aA 8.28	19.63aB 5.02
20–40	12.89aA 2.31	0.63bB 0.10	10.00aB 1.33	34.68aB 12.20	61.35bA 11.24	17.73aA 5.96
40–60	10.22bA 1.04	0.62bB 0.13	9.15aB 0.91	25.94bB 5.91	58.68bB 4.82	13.66bA 1.80
60–80	12.56aA 2.21	0.42cB 0.04	7.76bA 0.58	21.91bB 3.04	45.33cB 5.85	11.05bA 2.21
Disturbed Primary Semideciduous Seasonal Forest (DPSF)						
0–20	11.21aB 0.76	0.47aC 0.03	2.37bC 0.31	54.71aAB 4.49	35.25aB 2.65	9.32aAB 1.60
20–40	10.64bA 0.93	0.34bC 0.03	2.83bC 0.39	55.53aAB 4.93	30.81bB 2.85	8.28aB 1.54
40–60	8.00cAB 0.60	0.27cC 0.01	3.27aC 0.47	49.29aAB 4.79	23.88cB 2.67	7.35bB 1.46
60–80	8.67dAB 1.07	0.25dC 0.01	3.68aC 0.49	40.95AB 3.83	19.57dB 2.65	9.55aB 1.77
80–100	9.83bA 1.12	0.25dA 0.01	3.75aA 0.50	38.94bA 4.32	15.71eA 2.04	9.98aA 1.73
Disturbed Secondary Semideciduous Seasonal Forest (DSSF)						
0–20	10.86aB 1.03	0.36aC 0.02	1.58bD 0.29	97.84aAB 12.27	26.38aB 6.15	6.14bAC 1.37
20–40	11.11aA 1.10	0.29aC 0.02	1.72bD 0.30	95.53aAB 11.88	21.54bB 4.89	6.22aB 1.68
40–60	9.08aAB 1.07	0.26aC 0.02	1.92aD 0.38	81.59aAB 11.90	20.21bB 5.00	5.32bB 1.54
60–80	6.32bAB 0.84	0.22aC 0.02	1.98aD 0.58	51.28bAB 7.53	15.69bB 5.67	7.39aB 1.98
80–100	5.51bB 0.84	0.22aA 0.02	1.79aB 0.53	51.87bB 8.85	11.70cA 4.51	7.02aA 2.08
Transitional Forest to *Cerrado* (TFC)						
0–20	7.73aB 1.16	0.29aD 0.03	0.55aE 0.05	137.49aC 11.06	11.79aC 3.80	4.65bC 0.97
20–40	8.16aA 0.51	0.22bC 0.01	0.46bE 0.03	96.19bAB 10.89	3.82bC 0.93	4.41bC 0.33
40–60	6.70bAB 0.51	0.19bD 0.03	0.41bE 0.03	45.70cC 2.73	2.22cC 0.43	5.20aB 0.72
60–80	6.10bAB 0.53	0.20bC 0.02	0.37bE 0.02	34.32dC 3.09	1.80cC 0.33	5.75aC 0.91
80–100	5.04cB 0.46	0.20bA 0.02	0.35bC 0.03	36.80dC 6.19	1.57cB 0.30	5.12aB 0.62

Legend as explanations as in Table 1.

### Soil organic carbon stocks (SOCs)

Table 3 reports the SOCs values, for each of the five investigated BAF fragments, also comparing the used methodologies for SOC quantification.

**Table 3:** Comparisons in SOC stocks (SOCs) according to investigated BAF’s fragments and used method for SOC stock quantification (*CHNa* CHN analyzer, *WBm* Walkley–Black method).

BAF fragments	CHNa (t ha^−1^)		WBm (t ha^−1^)		≠ CHN *vs* Walkey (%)
	SOCs	CO_2_e	SOCs	CO_2_e	Total C and CO_2_e
LPSF	242.8 ± 22.9a	891.0 ± 84.0a	213.3 ± 23.4a	782.8 ± 86.0a	> 14
LSSF	257.6 ± 31.4a	945.2 ± 115.1a	216.5 ± 22. 7a	794.4 ± 83.2a	> 19
DPSF	176.1 ± 14.2b	646.3 ± 52.1b	88.5 ± 10.8b	324.7 ± 39.8b	> 99
DSSF	127.0 ± 18.3b	466.2 ± 67.2b	70.2 ± 11.4b	257.5 ± 41.9b	> 81
TFC	98.5 ± 6.0b	361.6 ± 21.9b	54.8 ± 3.1b	201.2 ± 11.5b	> 80
Mean values	180.9 ± 18.6a	662.0 ± 68.1a	128.6 ± 14.3b	472.1 ± 52.5b	> 40

Legend as in Table 1. Different letters, along rows and columns, showed significant statistical differences (p < 0.05).

The largest difference in SOCs amounts was observed for DPSF (Degraded Primary Semideciduous Seasonal Forest) with values of SOC extracted by CHNa 99% higher than SOC values obtained with the WBm. The smallest differences were observed for both investigated Late Forests (LPSF: 14%, LSSF: 19%). Soil organic carbon stocks and CO_2_e, when quantified through the CHNa, were 40% higher on average, considering all investigated physiognomies to 1 m soil depth, when compared with the WBm.

As previously observed (Table 3), the WBm showed a mean value of 128.6 ± 14.3 t ha^−1^ of SOCs stocked up to 1 m soil depth. Of this, 62% (79.7 t ha^−1^) was concentrated in the first 0–40 cm, while the remaining 38% (48.9 t ha^−1^) was in the 40–100 cm soil depth. By using CHNa extraction, SOCs mean values up to 1 m depth were 180.9 ± 18.6 t ha^−1^ (Table 3). In this case, 58% (104.9 t ha^−1^) was in the first 0–40 cm soil depth, while the remaining 76.0 t ha^−1^ (42%) was found in the last 40–100 cm soil depth. Table 4 shows SOC values according to the method of SOC measurement, BAF fragment, and investigated soil depth. Looking at the SOC content *vs* soil depth, considering all investigated BAF fragments, an inverse correlation was observed, with very similar behaviors for both applied SOC extraction methodologies, even if the WBm resulted in a lower estimate of SOCs compared to CHNa. Also in this case, the LPSF and LSSF fragments had the highest (p < 0.05) SOC contents, regardless of the investigated depth. The DPSF, DSSF, and TFC do not show statistically significant differences except when SOC was analyzed through CHNa. Indeed, in this case, at least for the soil depth to 60 cm, a clear statistical difference was observed, with the following trend in SOC contents: LPSF ≥ LSSF >> DPSF ≥ DSSF >> TFC. Soil organic carbon stocks at the 80–100 cm depth were three (CHNa) to two (WBm) times lower compared to the surface A horizons (0–20 cm).

**Table 4:** SOC content according to investigated BAF fragment, soil depth, and applied method.

BAF fragment	Depth (cm)	WBm (%)	CHNa (%)
LPSF	0–20	4.02aA	4.81aA
	20–40	3.29aA	3.72aA
	40–60	2.37bA	2.77bA
	60–80	1.94bA	2.16bA
LSSF	0–20	3.70aA	4.17aA
	20–40	2.87bA	3.33aA
	40–60	2.45bA	3.24aA
	60–80	1.78bA	2.44bA
DPSF	0–20	1.63aB	2.48aB
	20–40	1.03bB	1.62bB
	40–60	0.73bB	1.17cB
	60–80	0.52bB	0.95dB
	80–100	0.49bB	0.94dA
DSSF	0–20	1.57aB	1.82aB
	20–40	0.97bB	1.35aB
	40–60	0.66bB	1.05bB
	60–80	0.24cB	0.58cB
	80–100	0.19cC	0.50dB
TFC	0–20	1.01aB	0.92aC
	20–40	0.62bB	0.78aC
	40–60	0.42bB	0.64aC
	60–80	0.35bB	0.58bB
	80–100	0.33bB	0.52b B

Legend as in Table 1. Different capital letters show a significant difference (p < 0.05) among investigated BAF fragment; different lowercase letters show a significant difference (p < 0.05) among investigated soil depth.

### Factor analyses

The factor analyses (FA, Table 5) extracted a three-component model of robust statistical performance (all eigenvalues are over 1). The three extracted Factors explain 80% of the overall variance, meaning high statistical reliability. The first factor (F1) explained 56% of all observed variance in the data. F1 was positively concordant with pH, SOM, C-CHN, N, P, all basic exchangeable cations (K^+^_,_ Ca^2+^, Mg^2+^), CEC, BS, Cu, Mn, and both SOCs (regardless of the used method), while Al^3+^ was negatively correlated. F2 (14%) was positively correlated with SOM, C-CHN, Fe, and both SOCs. Finally, F3 (10%) showed BD as inversely correlated to C-CHN, N, K+, S, B, and both SOCs.

**Table 5:** Factor loadings of a factor analysis (n = 425); Extraction Method: principal factor analysis (PFA); Rotation Method: Varimax; bold loadings > 0.4.

	F1	F2	F3
pH	**0.892**	− 0.042	0.146
BD	− 0.351	− 0.174	− **0.624**
SOM	**0.449**	**0.664**	0.230
C-CHN	**0.596**	**0.461**	**0.524**
N	**0.499**	0.108	**0.600**
P	**0.845**	0.265	0.184
Al^3+^	− **0.856**	0.024	− 0.090
K^+^	**0.680**	0.046	**0.455**
Ca^2+^	**0.944**	0.136	0.219
Mg^2+^	**0.920**	0.185	0.178
CEC	**0.768**	0.394	0.220
BS	**0.947**	0.154	0.218
S	− 0.026	− 0.047	**0.680**
B	0.122	0.293	**0.766**
Cu	**0.749**	0.162	0.018
Fe	− 0.053	**0.840**	0.128
Mn	**0.758**	0.202	0.236
Zn	0.106	− 0.017	0.115
SOCs-WBm	**0.449**	**0.474**	**0.430**
SOCs-CHNa	**0.583**	**0.664**	**0.538**
Eigenvalues	11.180	1.990	1.566
Proportional variance (%)	56	14	10
Cumulative variance (%)	56	66	74

Significant values are in bold.

### Principal component analyses

**Figure 1:**
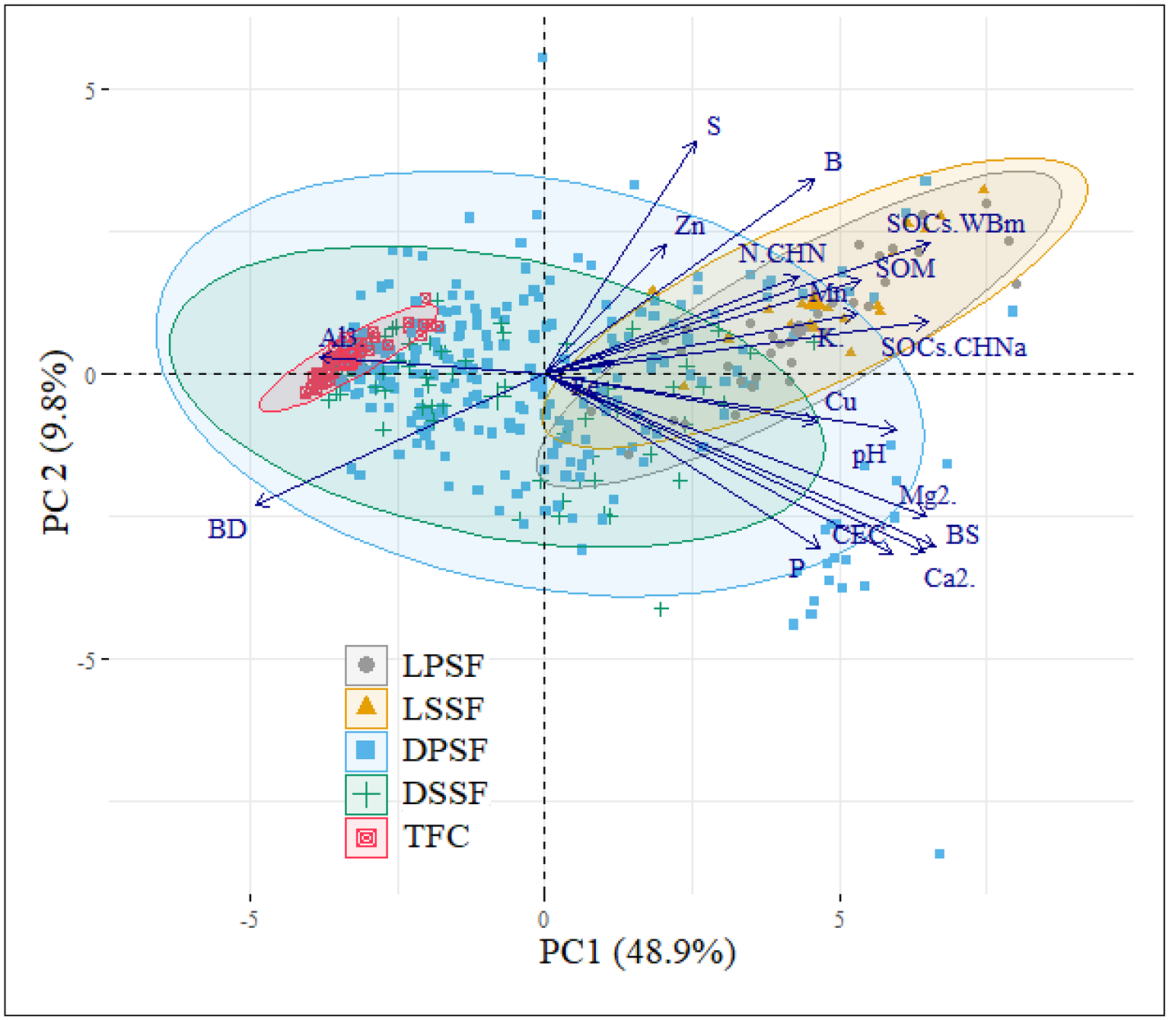
Biplot of a principal component analysis.

Figure 1 shows the biplot from PCA. Overall, the two main components explain alone the 69% of all observed variability. All investigated soil parameters (arrows) are over 1, thus showing a high statistical significance inside the model. The five mains investigated BAF’s physiognomies (points rounded by areas underlined by different colors) are grouped as: LPSF, LSSF ≠ DPSF, DSSF ≠ TFC.

## Discussion

### Soil physical–chemical features

Features of investigated soils among the five different BAF fragments show interesting similarities and differences. In most of the analyzed soil physical–chemical properties, we observed that LPSF, LSSF ≠ DPSF, DSSF ≠ TFC. Regarding human effects, past disturbances played a pivotal role in creating such clear differences. As argued by Roder et al.^27^, in more natural conditions like the LPSF and LSSF in this study, soils develop under mature forest vegetation, with higher spatial canopy cover and a well-developed organic surface horizon (O). Thus, O horizons can release basic cations in greater amounts, increasing soil pH^44^, while the forest canopy intercepts precipitation and limits leaching processes, thus limiting the loss of more soluble nutrients (basic cations)^45^. Conversely, environments like the TFC, representing the most disturbance, have less developed vegetation cover with a following enhancement of leaching processes and, consequently, decrease in soil pH. Pearson correlation coefficients (Supplementary Material 1) clearly confirmed such a behavior, showing a strong positive correlation between pH *vs* SOM (r = 0.62**), CEC (r = 0.53**) and, consequently, all basic cations (K^+^, r = 0.61**; Ca^2+^, r = 0.70***; Mg^2+^, r = 0.72***); and an inverse correlation was observed for SOM *vs* Al^3+^ (r =  − 0.35**).

Soil macro (N and P) and micronutrients (S, B, Cu, Fe, Mn, and Zn) showed a clear decreasing trend from LPSF/LSSF to TFC, i.e., from more preserved natural environments to less preserved ones. Soil nutrients also correlated to SOM (N: r = 0.50**, P: r = 0.43**, S: r = 0.37**, B: r = 0.65***, Cu: r = 0.44**, Fe: r = 0.27**, Mn: r = 0.70***, Zn: r = 0.32**), confirming previous studies showing SOM as the main source of such elements in tropical acidic and dystrophic pedosystems^9,10,27,46,47^.

Results from the investigated soil physical–chemical parameters show the pivotal role of human disturbance as a driving-factor in affecting pedoenvironment dynamics and behaviors. Even if pedoenvironments like those featuring LPSF and LSSF and somewhat even DPSF and DSSF are characterized by high to moderate resilience, with most of the investigated parameters improving as human disturbance decreased, recovery seems to be highly time-dependent. Indeed, the best soil conditions were found in those BAF fragments with no history of deforestation, exploitation for livestock, or agriculture, at least during the last century (LPSF), while the worst in the TFC, where humans acted as driving force until recently (30–40 years ago). Such observations suggest an efficient but highly time-dependent, slow recovery of BAF ecosystems from human disturbance, underlying the importance of their conservation and recovery.

### Soil organic carbon stocks (SOCs)

The carbon concentrations measured using CHNa were significantly higher than those measured using WBm, with mean values of 180.9 ± 18.6 t ha^−1^ and 128.6 ± 14.3 t ha^−1^ respectively. On average, CHNa measurements of SOC were 40% higher than WBm, with the greatest difference in the DPSF plots, where SOC was 99% higher with CHNa. Such results demonstrate that CHNa can play a pivotal role in detecting significant differences in SOCs values among different environments. The Walkley–Black method is less able to quantify SOC, and thus may under- or over-estimate SOC change when comparing between disturbed environments or between different management strategies. This outcome is in line with studies conducted worldwide that reported an underestimation in SOC values by using WBm compared to CHNa^21,48–51^. This is an intrinsic problem of the WBm, since it exhibits great variability in the dichromate oxidation degree and efficiency depending on factors such as SOC composition, soil bio-chemical-physical features, land cover and use, investigated soil horizon, etc.^47–52^. The CHNa detected higher SOC concentrations than the WBm, especially in soil horizons of BAF fragments with higher C content (Tables 1, 2). Similar results between the two methods were observed in lower C content conditions, as for the TFC or for deeper soil horizons. The decreased efficiency of the WBm compared to CHNa can be explained by the well-known affinity between iron-enriched tropical soils for OC^53^. Thus, CHNa was a more reliable method than WBm, particularly in soil horizons with larger SOCs due to mineral adsorption processes. Another factor could be associated with past land uses. Indeed, Davis et al.^54^ reviewed the SOC methods for tropical soils of Brazil, showing that in more anthropized or less natural areas (like those featured by relatively recent fires) WBm resulted in smaller and more variable results, especially for soil surface mineral A and Ap horizons. Thus, the authors concluded that underestimation of SOC content in such soils should be expected with WBm measurements. Segnini et al.^55^ and Tivet et al.^56^ confirmed that in the tropical soil of Brazil, WBm strongly underestimated SOC contents; Tivet et al.^56^ also identified historical land use as a factor affecting the efficiency of WBm.

Due to the concomitant combination of several issues (vide supra), conversion factors are extremely site-specific^56^, thus demonstrating the need to develop models calibrated for each investigated pedosystem to achieve a reliable comparison among SOC stocks. Despite SOC underestimation, WBm was the only method available to researchers in many cases; consequently, developing site-specific correction factors represents a strategy to improve data quality. Researchers worldwide proposed the application of a large range in correction factors (from 0.09 to 2.21) to increase WBm reliability^51^. A contradiction arises from the literature that authors often report the need to use CHNa to calibrate WBm measurements; this was done by sending samples outside investigated countries with related costs^22^. So, even if it is still widely used as a relatively low-cost method, it frequently requires a review/calibration process due to the large variability in the conversion factor. For instance, Gessesse and Khamzina^22^ revealed that in soil samples collected in several locations in Ethiopia, the most common correction factors did not improve the reliability of WB *vs* CHNa derived results. Thus, they proposed using the Bland and Altman method^57^, commonly applied in clinical research, for assessing the conversion factor, concluding that a correction factor of 1.32 for non-calcareous, carbon-poor Ethiopian soils can be considered reliable. This research, as with many others^58–66^ (vide supra), confirmed that correction factors are extremely site-specific and, thus not easily applicable without a double-check control with more expensive and sophisticated laboratory tools. As concluded by the review of Pribyl^67^, any factor used for OC conversion in SOM cannot be assumed as a universal constant; indeed, such numbers may be influenced by a combination of many factors (vide supra) that have the potential for serious error. Consequently, even if in the absence of other alternatives WBm often remains the only possibility to assess SOC, it is urgent to find new solutions that increase the reliability of data in unfavored socio-economic conditions. From this point of view, participatory and networking-based research promoted by Universities, Research Centers, Governments, and other institutional bodies such as non-governmental organizations (NGOs), represent opportunities to remedy this gap in data reliability. Implementing such kinds of projects is not only of scientific importance but also a moral question^22^. Indeed, GHG emissions, prevalently produced by developed countries, are responsible for climate change, mainly affecting developing ones^68^.

Looking at the SOCs *vs* soil depth, the LPSF and LSSF forests showed the highest values in all investigated depths compared to the other BAF fragments. In particular, if compared to the TFC (the most degraded fragment or the least natural one), LPSF and LSSF stocked around 4.5–5 times more SOC across each depth for both CHNa and WBm methods. The DPSF and DSSF seem to represent an intermediate stage between the aforementioned physiognomies. Depending on the measurement method (CHNa or WBm), DPSF and DSSF contained 2.5–3.5 times less SOC than LPSF and LSSF, but contained 1–1.5 times more than TFC.

Our results also show the deficiency in sampling and investigating the first 0–30 cm to quantify SOC^5^, which is the IPCC suggested minimum sampling depth for SOCs quantification. As argued by Jandl et al.^9^, considering only surface soil can dramatically underestimate overall SOC quantification, and sampling activities to at least 1 m soil depth can reveal additional important information. The present research confirmed that: (i) the SOCs between 40 and 100 cm were only slightly lower (≤ 10%) than stocks found in the first 0–40 cm; (ii) by extrapolating SOCs data from 30 to 100 *vs* 0 to 30 cm soil depth, the amount is significantly comparable, confirming Muños-Rojas et al.^69^ suggestion of soil sampling up to 1 m depth for SOCs quantification. Indeed, if SOC stock into the 30–100 cm soil depth was not measured, up to 50% of overall SOC (along the soil profile) would not be quantified.

We observed a clear increasing trend with decreased human disturbance level in both SOC stocks and CO_2_e amounts. As argued by Chenu et al.^70^, under most preserved environmental conditions, SOC stock can approach a long-term equilibrium, with SOM inputs coming from vegetation and animal residues, roots and their exudates, etc., balanced by its degradation from mineralization processes. This equilibrium can be shifted, as observed in the investigated areas, by changes in land management. Our results agree with Roder et al.^27^, which demonstrated a decrease in SOM as human disturbance increased in fragmented BAF. Souza et al.^47,71^ demonstrated that BAF recovery resulted from a large increase in SOCs after stopping human disturbance. In the present study, we demonstrated that such an increase in SOM was particularly found in Late Primary Semideciduous Forest (LPSF) and Late Secondary Semideciduous Forest (LSSF), which contained a significantly higher amount of SOCs compared to Disturbed Primary Semideciduous Forest (DPSF), Disturbed Primary Semideciduous Forest (DSSF), and the Transitional Forest to *Cerrado* (TFC). Previous research by Britez^72^ confirmed that among BAF physiognomies, the Disturbed Semideciduous Seasonal Forest can be characterized by low SOCs stock (between 31.7 and 37.5 t ha^−1^ up to 100 cm soil depth), especially in the case of relatively recent human disturbance. The results from our present research validate such a hypothesis, clearly showing a decreasing SOCs trend as DPSF → DSSF, i.e., at increasing human disturbance.

### Multivariate statistics

The FA and PCA combine soil physical–chemical features and SOCs, providing a more complete and complex perspective by quantifying relationship with factors other than human disturbance and depth. Furthermore, these models allow hypotheses about mechanisms involved in SOCs behavior compared to pedoenvironmental features to be explored.

### Factor analyses (FA)

Factor 1 (F1) shows that more natural, well-preserved fragments, i.e., the LPSF and LSSF, which feature a higher amount of SOM, thanks to a more developed forest cover and species complexity, are also characterized by (i) higher macronutrient contents (N and P), and (ii) higher CEC, exchangeable cation, and % base saturation, thus reflecting overall higher soil fertility. Under these conditions of high naturality, SOCs increased, with the CHNa method showing a higher statistical correlation than the WBm, confirming its higher analytical reliability. F1 also showed that increasing SOM led to a significant increase in Cu and Mn soil availability, which is consistent with SOM being one of the most influential factors modulating macro- and micro-nutrient availability in tropical soils^2^. Factor F1 can be explained as the *influence of BAF naturality in stocking higher amounts of SOC thanks to favorable pedoenvironmental conditions*. Factor F2, confirmed the strong relationship between SOM and SOCs, showing that at increasing BAF fragments naturality, we observed an increase in SOM and consequently stocked SOC. This factor adds to the pivotal role played by iron, showing that it increased at increasing SOM amounts. This was due to the well-known processes of SOM accumulation enhanced by organo-mineral interactions in tropical soils, with iron oxides playing an important role in stabilization^73^. Thus, F2 can be explained as the *Fe vs SOM interaction*. Factor F3 showed that at increasing bulk density (BD), i.e., soil depth (vide supra), a decrease in SOC and consequently soil macro (N), micronutrients (S, B), and exchangeable cations (K+), was observed. Since we know that such an increase in BD along soil depth follows the TFC > DPSF, DSSF > LPSF, LSSF trend, i.e., is inversely correlated to the BAF fragment conservation state, this factor can be explained as the *key role of BD in indicating BAF conservation state*. This interpretation is consistent with previous studies^74,75^, which showed that mean bulk density increased with soil depth, with this process being particularly enhanced by passing from well-preserved to degraded areas. Pontes et al.^76^ argued that human activities increased soil BD while decreasing porosity and creating a hostile edaphic environment for plant roots. Consequently, after human disturbance ends, the regeneration processes will be affected by BD initial conditions and the time required for establishing good edaphic conditions for plant roots. As the first pioneer species started colonization, porosity improved, creating more favorable conditions for hosting species from later successional stages. During this process, which requires a long time depending on starting conditions, BD will decrease, thus indicating improved conditions in the time-dependent steps toward BAF recovery.

### Principal component analyses (PCA)

**Figure 2:**
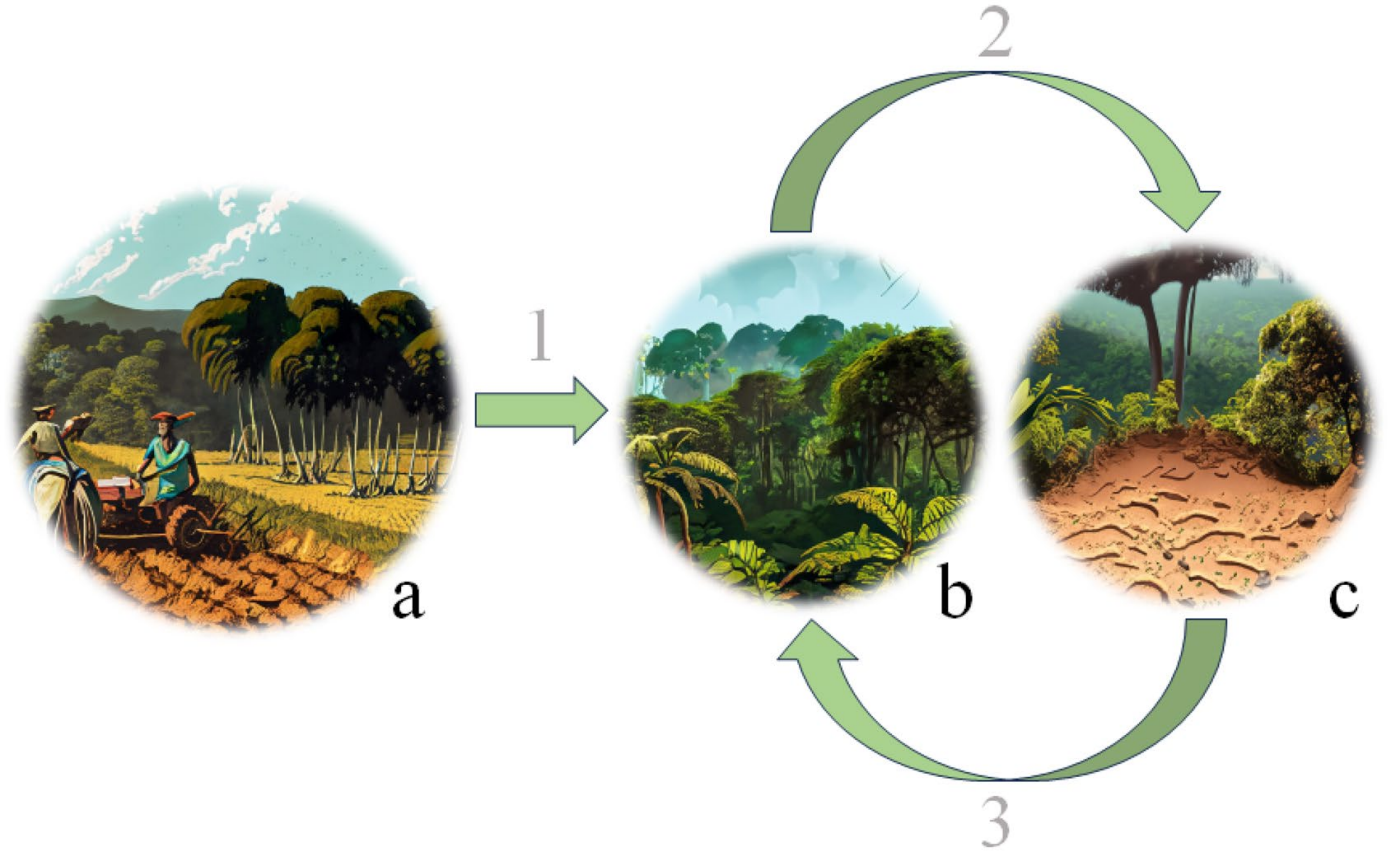
Soil-vegetation feedback in BAF fragments (generated using Adobe Firefly).

The PCA helps to visualize the FA outcomes. The five investigated BAF fragments are distinctively grouped with the greatest difference between group centroids along the first principal component (Fig. 2). Soil samples collected from the most developed, natural BAF physiognomies, i.e., LPSF and LSSF, form a distinct group along the first principal component. Arrows (indicating soil physical–chemical parameters) showed that these environments correlate with increased SOM and, consequently, SOC stocks and related extraction methods, together with N and B that are typically macro- and micronutrients in well-structured Atlantic Forest pedoenvironments^27^. The worst preserved BAF fragment (TFC) is located on the opposite position of LPSF and LSSF, visually confirming that soil samples collected in this environment have lower levels of all measured parameters besides bulk density. This environment is mainly influenced by Al^3+^ and BD, i.e., by acidic and more compacted soils. Again, PCA also confirmed that DPSF/DSSF physiognomies represent a transitional stage between highly-preserved (LPSF and LSSF) and most degraded (TFC) BAF fragments. Samples collected in DPSF and DSSF environments form two distinct groups located along the passage from TFC to LPSF and LSSF. The most important parameters in these environments are mainly soil micronutrients, which are pivotal in enhancing forest development towards more stable conditions^27^.

Overall, soil development and conservation features are strongly influenced by vegetation/forest conditions and vice versa, thus showing a positive feedback relationship (Fig. 2). As human activities (Fig. 2, number “1”) with related disturbance end (Fig. 2, letter “a”), after a variable period of time (depending on human disturbance intensity and years passed after last activities), vegetation start a relatively slow process of recovery (Fig. 2, letter “b”). Such a process strongly improves (Fig. 2, number “2”) soil's overall physical–chemical properties (Fig. 2, letter “c”), including fertility and the amounts of SOC stocked in the entire profile. Indeed, as vegetation reaches higher maturity, canopy cover increases, which results in (a) increased organic material inputs and (b), the formation of a thick soil organic (O) horizon; factors (a) and (b) preserve SOCs from mineralization and leaching processes thus substantially increasing its reserves along the entire soil profile^10^. Indeed, shrub and tree species create more organic complexes and deeper root networks, up to 18/20 m^9,10^. Consequently (Fig. 2, number “3”), the overall soil–plant system improves over time with respect to nutrient exchange, overall fertility, quantity of SOCs, complexity, biodiversity, etc., until reaching a dynamic equilibrium representing the forest/soil climax. The duration to reach this climax is strongly dependent on human disturbance, including the number of years since anthropogenic activity ended and whether additional effects such as climate change affect forest growth and development patterns.

## Conclusions

Assessing soil organic carbon stocks (SOCs) is a pivotal step in the challenging research field of monitoring and evaluating GHG emissions associated with the soil-climate feedback cycle. These activities are based on field data collection and laboratory analysis, requiring reliable instruments. A great gap can exist in underdeveloped and developing countries due to resource constraints, and these same countries will experience some of the most severe impacts from climate change, including damages in both environmental and socio-economic terms. Additionally, all of these impacts are enhanced in tropical systems, which play a pivotal role in the global carbon cycle and, consequently, in climate behavior. Brazil represents a paradigmatic example of a previously undeveloped country that, in a relatively short time, completely changed its economy, including dramatic land-use changes, that had both positive and negative, direct and indirect consequences on SOC stocks and behavior. The Brazilian Atlantic Forest (BAF) has been one of the most damaged ecosystems, together with the Amazon, making research on it strategically important worldwide. Soil organic carbon stocks quantification showed that the use of CHN analyzer (CHNa) was highly reliable compared to the Walkley–Black method (WBm). We suggest promoting collaborations between under-equipped laboratories and research groups with high experience with CHNa use to increase network-based research programs, thus providing more reliable data. Additionally, in tropical soils SOCs should be evaluated to at least 1 m depth. A large amount of SOCs is not properly considered if only the first 0–30 cm are collected, which can make further uses of these data (for instance, for GHG emission models) unreliable. Considering investigated BAF physiognomies, representing fragments with different levels and recency in human disturbance, the LPSF and LSSF are the most developed and well-preserved, with soils showing the highest SOC stocks. The Transitional Semideciduous Seasonal Forest to *Cerrado* (TFC) has the least developed forest canopy and worst preserved soil conditions, thus showing the lowest SOC stocks. The DPSF and DSSF represent a transitional stage among the LPSF/LSSF and TFC, with SOCs values slightly lower than in LPSF/LSSF while higher than in TFC physiognomies. Multivariate statistics showed that the investigated environments feature a complex dynamic feedback where the influence of multiple parameters can play a pivotal role in determining differences and commonalities among recovering forests. Regarding management practices, we demonstrated that BAF requires a long time to fully recover its initial conditions after the end of human disturbance; consequently, preservation and conservation strategies should be privileged in remnant fragments.

## Methods

### Study area

**Figure 3:**
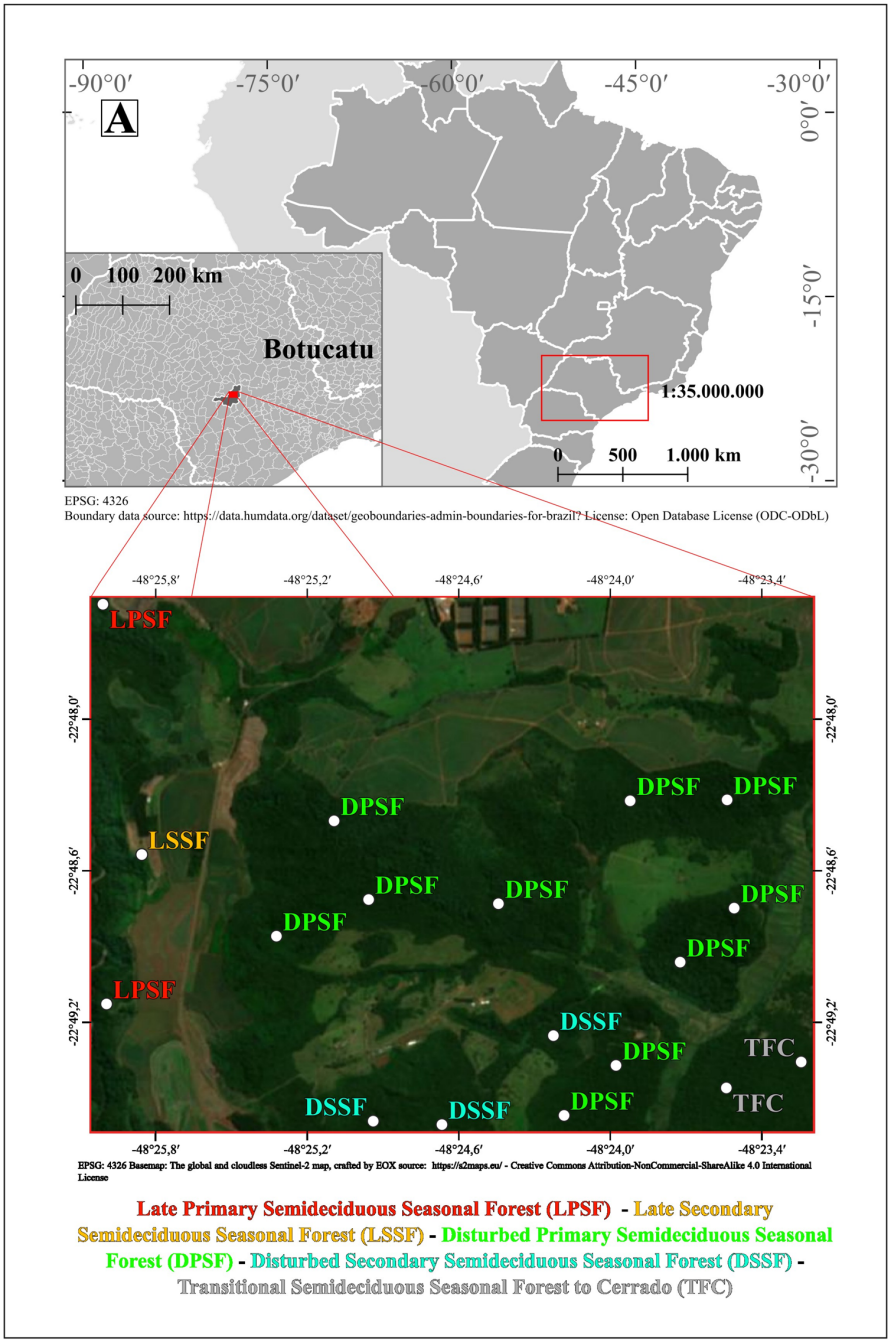
Study area (**a**) with plot distribution (**b**).

The research was conducted inside the “Edgardia and Lageado” (EL) experimental farms belonging to the Capivara River basin, Tietê Valley region, Botucatu Municipality in the south-east of Brazil (22° 47′ 30′′–22° 50′ S and 48° 26′ 15′′ to 48° 22′ 30′′ W; Fig. 3). The climate is classified as Cfa (Köeppen criteria^77^), i.e., subtropical which features hot temperatures (mean annual T: 20.6 °C) and high air humidity (mean annual P: 1707 mm).

The sites are located in the geological dominion of the western São Paulo Plateau, which is composed of igneous rocks. The research area is specifically a basaltic cuesta (from Spanish “slope”), which is a geological structure (technically an “homoclinal ridge”) with cliff/escarpment on one side and a dip/backslope on the other^78^.

Soils vary from low to medium–high fertility, depending on site overall features^27^. However, most are considered extremely fragile and under threat due to erosion processes enhanced by human activities. From a classification viewpoint^79^, Oxisols (deep Oxic Bo horizon) are the most widespread soil order. They are followed by “young” Entisols, passing to more developed Inceptisols (with a deep diagnostic Cambic Bw horizon). Alfisols and Ultisols (both featured by an Argillic Bt horizon) are present in small areas. All investigated soils had an udic moisture regime^27^.

### Experimental design

The vegetation cover greatly varied along the entire “Edgardia and Lageado” experimental farms. Indeed, as with the entire BAF over the last 500 years, the vegetation has been dramatically affected by anthropogenic disturbance and harvest, which is now found in fragmented patches with their own specific physiology and flora associations. For the purpose of classifying BAF fragments into a gradient of human disturbance, we performed an ad-hoc investigation of: (i) historical land-use changes compared using aerial and satellite images; (ii) physiognomic features; (iii) species composition; (iv) phytosociological associations; and; (v) ecological indicators, in addition to review of previous studies^27,80–82^. Based upon this investigation, we grouped sites into five main fragments, ordered from least to most human disturbance:

- *Late primary semideciduous seasonal forest (LPSF)* semideciduous seasonal forest are characterized by the occurrence of a seasonal climate (drought-cold winters-rainy summers) responsible for the foliage’s semi-deciduousness, i.e., partial leaf fall. The term late primary are used for indicating BAF fragments containing almost-native vegetation cover. Indeed, due to their location in not easily accessible areas, they experienced a very low level of human disturbance, with no history of deforestation, selective logging, livestock or agriculture, at least during the last century (Fig. 3a).
- *Late secondary semideciduous seasonal forest (LSSF)* represent a case of advanced regenerative LPSF phase, after a human disturbance (such as logging of forest species for economic purposes), usually occurring more than 30–40 years ago. The advanced regenerative processes (if compared to DPSF and DSSF, below) was favored by their not easily accessible location.
- *Disturbed primary semideciduous seasonal forest (DPSF)* these are BAF fragments containing less natural conditions compared to Late Primary forest (vide supra). Indeed, the forest structure is less developed due to more intensive selective logging at the beginning of the last century. Additionally, these fragments are located in more easily accessible areas.
- *Disturbed secondary semideciduous seasonal forest (DSSF)* represent a case of moderate regenerative DPSF phase. In particular, after a human disturbance such as logging of forest species, fire, and agricultural activities, occurring in a more recent time compared to DPSF.
- *Transitional semideciduous seasonal forest to Cerrado (TFC)* are those BAF fragments affected by a clear decrease in forest canopy cover due to selective logging, grazing, agricultural activities, etc., usually occurring more than 30–40 years ago. Even if the term *Cerrado,* indicates a specific Tropical savanna ecoregion, in the investigated area, it represents an ecotonal transition between BAF and itself^38^.

Thus, investigated areas are or have been affected by human influence, even if they are now considered “natural” or “seminatural” environments. Indeed, there has been continuous interaction and perturbation by humans throughout the history of the BAF^27^. In fact, there are no BAF fragments that have not been disturbed or influenced by human activities to some degree. Thus, when investigating such fragments, the history of human interaction and activities must be carefully incorporated since humans represent a driver force in the forest’s past, present, and future development and behavior^27^.

Eighteen 20 × 100 m (2000 m^2^) permanent plots (Fig. 3b, Supplementary Material 2) were randomly distributed in LPSF, LSSF, DPSF, DSSF, and TFC. The plot size and distribution were defined according to the methodology proposed by Roder et al.^27^. Specifically, the sampling unit and their distribution along the five investigated BAF fragments were selected after careful in-field study and GIS investigation carried out before the beginning of the research by considering the difference between: (i) the vegetation from several perspectives (vide supra); (ii) morphological and slope aspects; (iii) soil features; (iv) history in human disturbance; (v) area extension of each investigated kind of vegetation. Consequently, the 18 plots were distributed in numbers of 2, 1, 10, 3, and 2 in LPSF, LSSF, DPSF, DSSF, and TFC, respectively (Fig. 3b). Due to the previously reported factor, the uneven distribution was necessary to capture the whole variance among investigated environments. Milliren et al.^83^ assessed whether uneven plot distribution can bias random effect estimation and, consequently, observed variance and obtained outcomes, and concluded that applied plot distribution captured a truly random effect without affecting the statistical significance.

When possible, the edge effect was avoided by distancing 500 and 200 m from each plot’s and forest’s border, respectively. In some few cases (such as LPSF and LSSF), distances were closer due to the smaller size of the investigated fragments and the nearest presence of other environments. However, a minimum distance of 100 m was always applied, and soil and vegetation samples collected far from the border (vide infra). Replications consisted of subdividing the eighteen permanent plots (vide supra) into twenty subplots of 10 × 10 m (100 m^2^) (Supplementary Material 2). To completely randomize soil collection within the sampling unit, five subplots were randomly selected to take a composite sample (three sub-samples were collected from each sub-plots; note red points in Supplementary Material 2) from each, using the method defined by Husch^84^.

### Soil sampling and analyses

Within each of the five randomly selected subplots (vide supra), soil samples were collected from 5 soil depths, i.e., 0–20 cm, 20–40 cm, 40–60 cm, 60–80 cm, and 80–100 cm. In LPSF and LSSF plots, soil sampling stopped at 80 cm because these soils were less deep since they were located on steep slopes.

From each horizon, undisturbed soil mineral samples were collected with metal cylinders for bulk density determination^85^. Soil analyses were then performed in the laboratory on air-dried ⌀ < 2 mm sieved soil, as recommended by Brazilian official procedures^86^. Soil pH-CaCl_2_ and H + Al (potential acidity) were assessed potentiometrically with a glass electrode in a 1:2.5 1 N CaCl_2_ soil/solution mixture. Total N was analyzed using a CHN analyzer (dry combustion). Total P was analyzed through a NH_4_Cl/HCl acid digestion. Cation-exchange capacity (CEC) was assessed via saturation with BaCl_2_ (pH 8.2). Calcium and Mg were determined by extraction in 1 M KCl. Concentrations of soil micronutrients (Al, B, Cu, Fe, K, Mn, and Zn) were measured by the Olsen extraction method (pH 8.5).

Soil organic carbon was determined using two methodologies: (i) the Walkley–Black method; (ii) an elemental (CHN) analyzer (2400 Series II System, Perkin Elmer, US). In the (i) case, soil organic matter was obtained by multiplying C results for a conversion factor of 1.724 (assuming that SOM contains 58% of OC), commonly used and recommended by IPCC^5^ and, as reviewed by Kim et al.^19^, several authors regardless of the pedoenvironmental conditions.

Soil organic carbon stocks (SOCs, hereafter) were calculated as follows:

1 $$SOCs \, \left( {{\text{t ha}}^{ - 1} } \right) \, = \, HD \, \times \, BD \, \times \, SOC \, \times \, 10,$$

where, *HD* was the soil horizon depth (cm), *BD* was the bulk density (g cm^−3^), and *SOC* was the soil organic carbon content of the investigated horizon (%).

Total SOCs were converted into CO_2_equivalent (CO_2_e) using the conversion factor 3.67, i.e., by the ratio between carbon dioxide’s molecular mass and carbon’s atomic mass^87^.

### Statistical analyses

Statistics were done by using the software program R^88^. The analysis of variance (ANOVA) was conducted to assess statistical differences (p < 0.05) in soil C stocks in the following cases: (i) by using different methodologies (WBm *vs* elemental analyzer); (ii) when comparing the five different fragments; and, (iii) when comparing the five different investigated soil depths. A posteriori comparison was also done by using the Tukey-HSD (honestly significant difference) test at p < 0.05. In case of parametric requirements were not satisfied, the Kruskal–Wallis test was used at p < 0.05. Several multivariate statistical techniques were also used: (i) a factor analysis (FA) used for data interpretation and hypothesis testing; (ii) a principal component analysis (PCA) for data compression and visualization, without making any assumptions about the causal relationships among investigated variables. The procedure proposed by Reimann et al.^89^ and modified by Capra et al.^90^ for soil physical–chemical datasets, was applied for factor analysis (FA). In particular: (i) the entire dataset was tested for normal distribution; (ii) the raw datasets were Box–Cox transformed, thus approaching normality; (iii) a Pearson correlation matrix (CM) was constructed with the Box–Cox transformed data; and, (iv) FA was obtained based on the CM as elaborated in point (iii); (v) varimax rotation was applied for a more robust statistical approach and to facilitate the results’ interpretation. Principal component analysis (PCA) was implemented by using the robust procedure proposed by Filzmoser et al.^91^: (i) dataset was isometric log-ratio transformed (ilr); (ii) since the transformation results in uninterpretable variables in terms of their original names, data was; (iii) back-transformed to the centered log-ratio (clr) transformation to allow for an interpretation in terms of the original variable names.

### Supplementary Information

Supplementary Information 1.

Supplementary Information 2.

## Data Availability

The datasets generated during and/or analysed during the current study are available from the corresponding author on reasonable request.
