# Supplementary figures and images for: Evaluating carbon stocks in soils of fragmented Brazilian Atlantic Forests (BAF) based on soil features and different methodologies
Source: Sci Rep. 2024 May 1;14:10007. doi: 10.1038/s41598-024-60629-y (PMC11063065; doi:10.1038/s41598-024-60629-y)

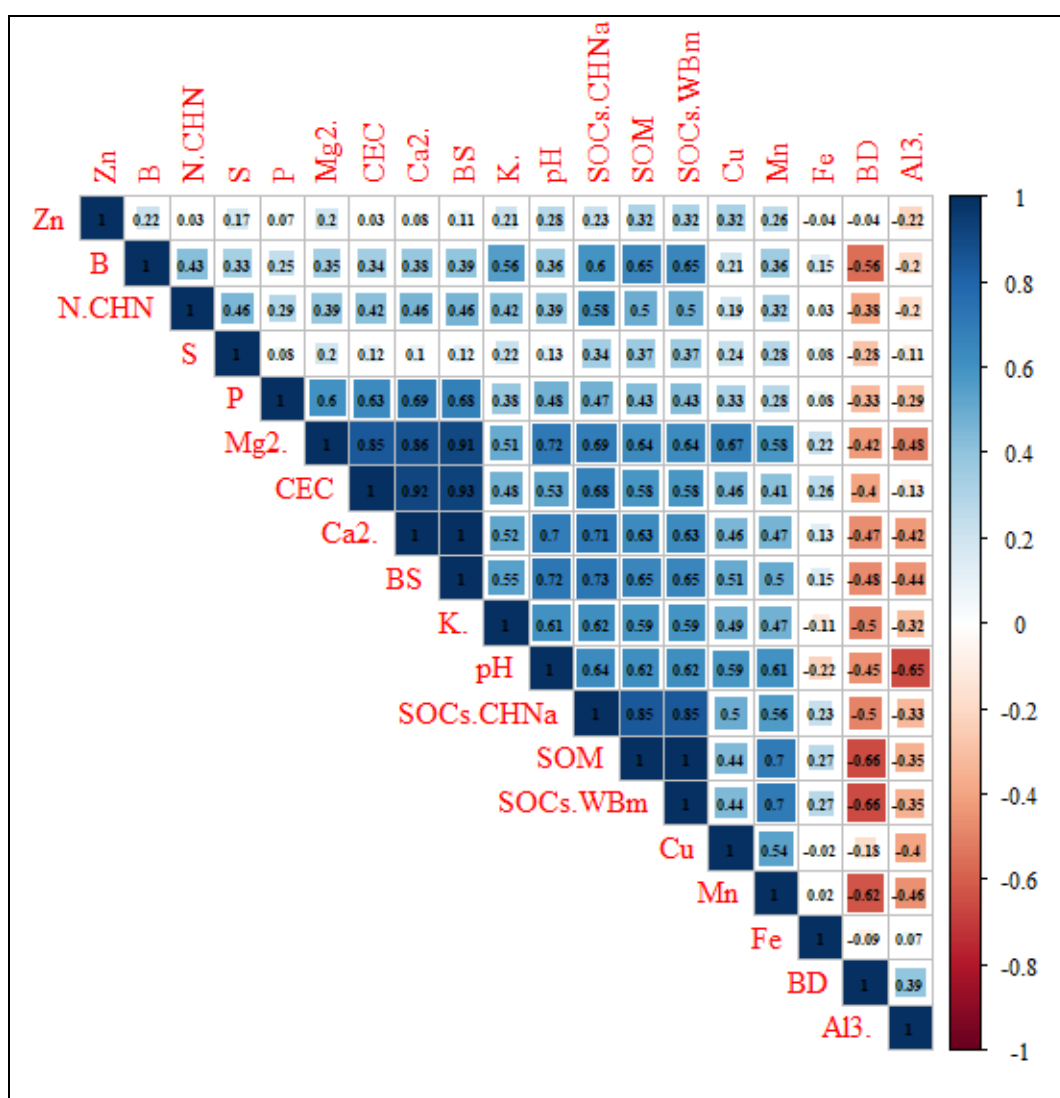

Supplementary Material 1. Pearson's correlation matrix.

Supplement: Supplementary file 1 — Supplementary Information 1. [file 41598_2024_60629_MOESM1_ESM.pdf]
