# Supplementary material for: Evaluating carbon stocks in soils of fragmented Brazilian Atlantic Forests (BAF) based on soil features and different methodologies
Source: Sci Rep. 2024 May 1;14:10007. doi: 10.1038/s41598-024-60629-y (PMC11063065; doi:10.1038/s41598-024-60629-y)
Supplement: Supplementary file 2 — Supplementary Information 2. [file 41598_2024_60629_MOESM2_ESM.pdf]

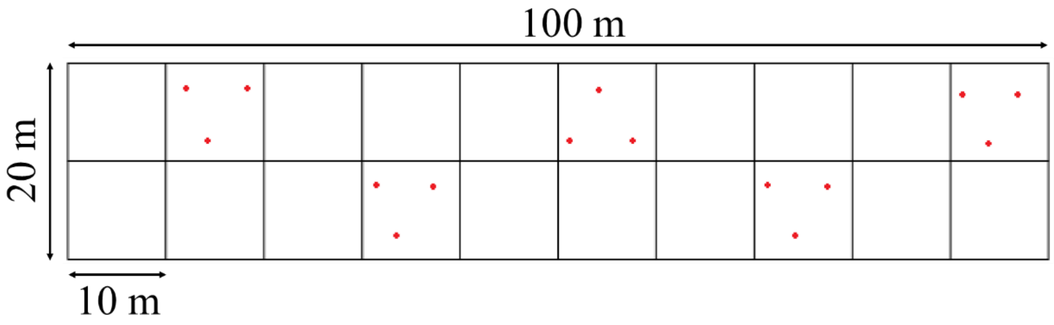

Supplementary Material 2. Plot (20 × 100 m) and subplots (10 × 10 m) scheme. Red points are for composite samples.
